# Detection of benign proliferative lesions on vocal cords with voice handicap index

**DOI:** 10.3892/etm.2012.645

**Published:** 2012-07-24

**Authors:** XUEKUN HUANG, GEHUA ZHANG, XIUJIN LIU, TAO WANG, LING ZHU

**Affiliations:** Department of Otolaryngology, Head and Neck Surgery, The Third Hospital, Sun Yat-Sen University, Guangzhou, Guangdong 510630, P.R. China

**Keywords:** benign proliferative lesions on vocal cords, voice handicap index, voice disease

## Abstract

The aim of this study was to investigate the significance and practical applicability of the voice handicap index (VHI) in the diagnosis of benign proliferative lesions of the vocal cords (BLVCs). The detection of VHI included the calculation of functional (F), physical (P) and emotional (E) domains, and the score of each domain and total score (TVH). The VHI was scored in patients with BLVCs and healthy controls. Eighty-four patients with BLVCs and 27 healthy controls were enrolled in the present study. The F, P, E and TVH scores were 10.40±7.84, 19.88±8.81, 9.39±8.49 and 39.37±21.83, respectively, in the BLVC group and 0.78±0.97, 0.85±1.06, 0.26±0.72 and 1.89±2.31, respectively, in the control group. A significant difference was found between the two groups (P<0. 01). The daily duration of speech and course of BLVCs did not correlate with the VHI score in BLVC patients (P>0.05). There was no marked difference in the VHI score between voice-consuming and non-voice consuming occupations (P>0.05) or between males and females (P>0.05). In BLVC patients, VHI may subjectively express the voice handicap, while daily duration of speech, course of BLVC, occupation and gender have no impact on VHI.

## Introduction

Voice diseases affect the quality of voice and the function of vocal cords, and cause psychological and social problems to various extents, thus affecting the quality of life. The voice handicap index (VHI), first proposed by Jacobson *et al* (1) in 1997, is an effective parameter reflecting the effect of voice diseases on physiological, social and psychological functions. It has been the most common parameter in use for subjective self-evaluation of voice. The VHI has favorable internal consistency, repeatability and reliability, and reflects the subjective perception of the severity of the voice handicap (2). Xu *et al* (3) translated the scale for the detection of VHI into Chinese, and the scale of the Chinese edition was also demonstrated to possess favorable reliability and validity. Vocal cord polyps, nodules and cysts are common benign proliferative lesions of the vocal cords (BLVCs) and common causes of clinical voice handicap. These diseases are characterized by changes in the superficial lamina propria and epithelial layer. In the present study, 84 patients with BLVC were recruited and VHI was determined. The aim of this study was to explore the correlation between BLVC and VHI and investigate the clinical significance of VHI in BLVC.

## Patients and methods

### Ethics statement

The experiments were approved by the Administrative Ethics Committee of Sun Yat-Sen University (Guangzhou, China; SYSU-201002).

### Patients

Between June, 2011 and January, 2012, a total of 84 patients with laryngoscopically-proven BLVC were recruited from the Department of Otolaryngology, Head and Neck Surgery (Guangzhou, China). Of the 84 patients, 41 had vocal cord polyps, 41 had vocal cord nodules and 2 had cysts. There were 6 males and 78 females with a mean age of 31.85±8.36 years. The course of BLVC ranged from 1 to 408 weeks. In addition, 27 healthy controls were recruited and there were 8 males and 19 females with a mean age of 31.04±8.16 years (range, 20–53 years). The controls had normal voice and no history of throat diseases or voice handicap. The subjects in the two groups had no history of smoking or drinking and no history of respiratory or nervous system diseases.

### VHI scoring

The scale of the Chinese edition was used for the detection of VHI (3). The impact of voice handicap on the quality of life was divided into three domains; physical (P), functional (F) and emotional (E). In each domain, there were 10 items and patients scored each item as follows: score 0, no; score 1, less; score 2, sometimes; score 3, often; score 4, always. The sum of the scores of the 10 items served as the score of each domain (range, 0–40), and total VHI score (TVH) was the sum of scores in the three domains (range, 0–120). The higher the score or total score, the more severe the effect of voice handicap on quality of life or self-reported influence, respectively. The patients were evaluated with the VHI scale. The trained investigators explained the details to each patient and the manner in which to respond to questions. The patients truthfully responded to each question.

### Survey of disease condition

The daily duration of speech, course of disease, voice (teacher and salesperson) or non-voice (homemaker and farmer) consuming occupations were recorded.

### Statistical analysis

SPSS version 16.0 for Windows was used for statistical analysis. Comparisons between BLVC patients and controls were performed with the independent samples t-test and the correlation was evaluated using Spearman’s correlation analysis. P<0.05 was considered to indicate a stastistically significant difference.

## Results

### Comparison of VHI between BLVC patients and controls

The scores of VHI in the two groups are listed in Table I and statistical analysis revealed a significant difference between the VHI scores of the two groups (P<0.01).

### Correlation of VHI score with daily duration of speech and course of disease in BLVC patients

Among the BLVC patients, the daily duration of speech was 1–10 h and the course of BLVC was 1–408 weeks. The correlation coefficients of VHI with daily duration of speech and course of disease are listed in Table II. No significant correlation between VHI and daily duration of speech, as well as course of BLVC, was noted among the BLVC patients (P>0.05).

### VHI scores in BLVC patients with different occupations

Of the 84 patients with BLVC, 57 had voice-consuming occupations and 27 had non-voice consuming occupations (Table III). No significant difference in the VHI scores was found between patients with different occupations (P>0.05).

### VHI scores in female and male BLVC patients

Of the 84 patients with BLVC, 6 were male and 78 were female (Table IV). No marked difference in VHI was noted between the male and female BLVC patients (P>0.05).

## Discussion

In the VHI scale proposed by Jacobson *et al* (1), the score 0–30 was defined as an absent or mild voice handicap, 30–60 as moderate voice handicap and 60–120 as severe voice handicap. In the present study, the VHI scores in the BLVC group were markedly higher than those in the control group (P<0.01). The VHI score of BLVC patients was 39.37±21.83 in the present study, which was comparable to that (36.96±19.80) reported in the study of Rosen *et al* (4). This finding suggested that these patients self-reported a moderate voice handicap and VHI was a simple and effective tool to detect the effect of voice diseases on the physical, social and psychological functions.

As a method for the detection of self-reported voice handicap, certain factors may affect the self-evaluation. These include personality, stage of the disease, previous vocal function, occupation and social status (5). Thus, the self-evaluation of voice handicap varies among patients. In the present study, the daily duration of speech was 1–10 h in BLVC patients, which was not associated with the VHI score (P>0.05). In addition, the course of BLVC ranged from 1 to 408 weeks, which was also not associated with the VHI score (P>0.05). Behrman *et al* (6) observed that the VHI score in patients with a long course of disease was lower than that in patients with a short course, which was in contrast to our finding. This may be attributed to the differences in study population and methodo logy. Although voice-consuming occupations have a higher demand on voice than do non-voice consuming occupations, no marked difference in VHI score was noted between patients with different occupations (P>0.05). Our findings demonstrated that daily duration of speech, course of BLVC and occupation did not affect the VHI scores.

In the present study, the incidence of BLVC in females (n=78) was higher than that in males (n=6), which may be correlated with the higher tone and higher frequency of vocal cord vibration in females. These factors may cause damage to the superficial lamina propria and epithelial layer of the vocal cord. In addition, the hormone levels in females fluctuates over time (including menstrual period and time period subsequent to menopause), which may significantly affect the voice more than when compared with males. In the present study, no significant difference was observed in the VHI scores between males and females (P>0.05). This finding was similar to that of Behrman *et al* (6) who demonstrated that gender did not affect the VHI of BLVC patients. In certain studies, the incidence of voice diseases (including BLVC, vocal cord paralysis and functional dysphonia) in females has been confirmed to be higher than that in males, which may be due to females paying more attention to their voice and thus more actively receiving interventions (7).

The quality of voice may be evaluated subjectively (due to subjective perception of voice handicap or VHI) and objectively (voice acoustic analysis). The correlation between VHI and findings in voice acoustic analysis remains unclear. Schindler *et al* (8) suggested that the physical score of VHI in patients with vocal cord nodules was correlated with the jitter (Jitt), shimmer (Shim) and normalized noise energy (NNE). In addition, findings of other studies demonstrated the functional and emotional scores, but not the physical score, which in VHI were associated with Jitt and Shim (9). Thus, the subjective perception of voice handicap cannot be replaced and predicted with findings in objective detection.

Our results demonstrate that the influence of BLVC on the physical, social and emotional functions may be detected via self-evaluation, and the VHI score in BLVC patients is not correlated with the daily duration of speech, course of BLVC, occupation or gender. However, the vocal functions are multidimensional and single detection only reflects the effect in one dimension. Thus, in clinical practice, comprehensive methods should be employed to evaluate the voice functions.

## Figures and Tables

**Table I:** Scores of VHI in BLVC patients and controls (mean ± SD).

Group	F	P	E	TVH
BLVC (n=84)	10.40±7.84^a^	19.88±8.81^a^	9.39±8.49^a^	39.37±21.83^a^
Control (n=27)	0.78±0.97	0.85±1.06	0.26±0.72	1.89±2.31

a P<0.01 vs. control group. F, functional domain; P, physical domain; E, emotional domain; TVH, total VHI score; VHI, voice handicap index; BLVC, benign proliferative lesions of the vocal cords.

**Table II:** Correlation coefficients of VHI with daily duration of speech and course of BLVC among BLVC patients.

VHI	Daily duration of speech	Course of BLVC
F	0.139	0.073
P	0.163	0.031
E	0.018	0.073
TVH	0.107	0.141

F, functional domain; P, physical domain; E, emotional domain; TVH, total VHI score; VHI, voice handicap index; BLVC, benign proliferative lesions of the vocal cords.

**Table III:** VHI scores in BLVC patients with voice and non-voice consuming occupations (mean ± SD).

Group	F	P	E	TVH
Voice-consuming occupation (n=57)	11.09±8.16	21.07±9.11	10.28±8.92	41.98±22.38
Non-voice consuming occupation (n=27)	8.96±7.04	17.37±7.70	7.52±7.32	33.85±19.88

F, functional domain; P, physical domain; E, emotional domain; TVH, total VHI score; VHI, voice handicap index; BLVC, benign proliferative lesions of the vocal cords.

**Table IV:** Scores of VHI in male and female BLVC patients (mean ± SD).

Group	F	P	E	TVH
Males (n=6)	8.50±5.39	15.17±4.83	4.33±4.89	28.00±13.46
Females (n=78)	10.55±8.00	20.24±8.96	9.78±8.61	40.24±22.16

F, functional domain; P, physical domain; E, emotional domain; TVH, total VHI score; VHI, voice handicap index; BLVC, benign proliferative lesions of the vocal cords.
